# Community engagement for artificial intelligence health research in Africa

**DOI:** 10.12688/wellcomeopenres.23684.1

**Published:** 2025-03-20

**Authors:** Harriet Nankya, Debra Mathews, Kadija Ferryman, Odia Kane, Joseph Ali

**Affiliations:** 1 Philosophy, Makerere University College of Humanities and Social Sciences, Kampala, Central Region, Uganda; 2 Johns Hopkins University Berman Institute of Bioethics, Baltimore, Maryland, USA

**Keywords:** Africa, Artificial Intelligence, Community Engagement, Research

## Abstract

Artificial Intelligence holds the potential to benefit communities in numerous areas, including health. Artificial intelligence health research is, among other things, advancing the accuracy of diagnosis, enabling new drug and treatment options, and reducing costs in healthcare. Like elsewhere, artificial intelligence health research is rapidly expanding across the African continent; however, numerous co-travelling ethical challenges - including those related to data protection, equitable access, and data colonization - are under-addressed. Community engagement is a process through which a number of pertinent health research ethics issues affecting communities can be identified and collaboratively pursued; however, there currently is limited understanding of the opportunities and challenges for community engagement in artificial intelligence research globally. In order to advance collective understanding and support policy and practice innovation, this paper interrogates how communities in Africa could be engaged in artificial intelligence health research. It provides a justification for community engagement in artificial intelligence health research, and discusses its application in African communities. It concludes by offering some context-specific recommendations for priority attention.

## Plain language summary

This paper addresses the role of Community Engagement (CE) in Artificial Intelligence (AI) health research in Africa. Authors reviewed existing literature and critically reflected on the topic from their perspectives as experts in bioethics, digital health, AI, and global health. Community engagement can enable the incorporation of community values, interests, and structures across all stages of the research process; however, the implementation of CE for AI health research in Africa is prone to face challenges. This paper analyzes how CE in AI health research in Africa ought to be conducted, and offers an initial set of context-specific considerations that need to be addressed in order to fully realize its potential.

We identified and reviewed published articles and reports that addressed pertinent features of AI health research, health research conducted in African communities, and CE in health research in Africa.

The findings indicate a particular need for CE in AI health research given its potential to have social implications. The conduct of CE in AI health research faces challenges in Africa including limited exposure and education about AI, gender disparities, and cultural values and practices that may not align with AI development and use. Context-specific priorities for CE in AI health research in Africa were identified.

These findings lay a groundwork for further research to guide policy and practice for CE that can advance the ethical development of AI for health in Africa.

There was limited published literature on the topic, this review also used non-systematic methods and relied significantly on the expertise of its authors in developing pertinent insights. There is a need for empirical research on the role of CE in AI health research, and to identify emerging practices.

## Introduction

Artificial Intelligence is a machine’s ability to perform some functions or tasks that are usually associated with human minds ^1^. AI encompasses a variety of technology functions including machine learning (ML) and natural language processing (NLP). AI holds the potential to benefit communities in numerous areas, including health. Further, AI holds great promise for healthcare and medicine worldwide, with the possibility of advancing diagnostic accuracy, enabling identification of new drug and treatment options, and reducing costs associated with accessing health information for some, among other potential benefits ^2, 3^.

The application of AI for health in Africa and elsewhere is, however, known to face ethical issues. These include how to explain the role and capabilities of algorithms, and challenges stemming from poor health data quality and data bias, inadequate privacy protections, limited transparency, and inequities in data collection, access and ownership that can concentrate the risks and de-localize the benefits of AI ^4– 7^. To put a finer point on this last concern, AI is built on data to provide the information needed to train AI models and to make accurate predictions. The acquisition, handling and use of these data could raise potential social risks such as discrimination and marginalization of populations and economies. The global data ecosystem is currently one in which certain actors, typically located in high-income countries, have great control over data systems ^4^. This potentially brings about imbalance wherein the powerful have the capacity to extract data from those with less power, without adequate sharing of benefits ^4^. This could further raise issues of social and distributive justice.

Community engagement is an important process through which certain ethical issues in research can be identified and addressed, especially those likely to impact the public ^8^. Through CE, community members are respected as rational and autonomous beings whose dignity, interest, concerns, values and structures are respected in the research. Much as CE stands to aid in identifying and addressing pertinent ethical issues in AI research, there is a need to interrogate its applicability, especially in the African context where CE has a strong tradition and AI is rapidly being introduced to advance public health and health research objectives. This paper is an effort to achieve that by defining what CE in research is, providing justification for CE in AI health research in Africa, identifying the challenges facing CE application for AI research in Africa, and the context-specific priorities for CE in AI health research in Africa.

### 1. What is community engagement in research?

To date, there is no commonly agreed upon definition of CE because there is no universally accepted definition of “community” or “engagement”. “Community” generally denotes a group of people that have a common identity or interest such as living in the same geographical proximity, having a common disease, sharing an ethnicity, and so forth. In the context of research, the term “engagement” often refers to a reciprocal relationship between the research team and the research community ^9^. While there is no standard definition for CE, this paper adopts Ahmed & Palermo’s definition of CE as the “process of inclusive participation that supports mutual respect of values, strategies and actions for the authentic partnership of people affiliated with or self-identified by geographic proximity, special interest, or similar situations to address issues affecting the well-being of the community of focus” ^10^.

The Joint United Nations Program on HIV/AIDS (UNAIDS) Good Participatory Practices (GPP) guidelines (2011) established principles on CE, which have been echoed by other guidelines and projects ^11– 13^ including the Uganda National Guidelines for Research Involving Humans as Research Participants. These Uganda guidelines identify relevant principles for CE as: “mutual trust,” “mutual respect,” “mutual understanding,” “accountability,” “integrity,” and “transparency” ^13^.

Mutual respect requires that both parties, that is the researchers and the community, identify and honour the perspectives and values of each other. Such respect would, among other things, establish a basis for effective communication which could yield mutual trust and opportunities to form and strengthen relationships. Building and maintaining trust among stakeholders requires an open, truthful, and active CE process.

Mutual understanding requires that both partners share openly and listen to each other, while respecting the information shared. This often is facilitated by adopting a presumption of epistemic legitimacy, where different ways of knowing the world are given equal face value. The researchers should be prepared to articulate clearly the goal of the research and to share other pertinent information with an openness to change. When potential misconceptions are identified, stemming from either the community or the researchers, there is often a need to “lean in” to those understandings and to seek out prior assumptions or knowledge gaps. Through this process, the community can also provide information about their priorities and expectations.

In a bid to ensure integrity, CE itself should conform to high scientific and ethical standards, following best-practices and ideally integrating some form of monitoring. This will help to elevate the status of engagement activities while laying a foundation for downstream research integrity.

To ensure transparency, CE requires honesty in communication and sharing of information that demonstrates conformity of actions with commitments. In this way, transparency is closely associated with accountability. Importantly, information should be shared in a format and context that is accessible, and fair procedures should be established for making decisions.

Lastly, accountability is required of all stakeholders involved in research, including the researchers, the community, funders, and those responsible for research oversight. This is essential not only to establishing trustworthiness of a research activity, but also to broader goals of ensuring stewardship of the research enterprise, which is itself a fragile social good. Engagement processes can provide one means through which accountability requirements are surfaced and monitored.

### 2. Justification for community engagement in health AI research in Africa

Calls for CE in research, including in AI research, often reflect a desire to broaden the historical ethics paradigms that have sometimes prioritized autonomy of the individual research participant to also include the community where those participants belong. Based on Gyekye’ ontology, an individual is a communal being; that is, she does not live in isolation but in communities ^14^. This necessitates an understanding of the social aspects of an individual being together with the ethical consideration of communities as a whole ^15, 16^. This understanding is consonant with conceptualizations of individual health as a state of well-being that includes due consideration of social determinants. In the health research context, protecting autonomy constitutes a protection of particular ‘first-generation’ civil and political rights such as privacy, safety, and welfare of individual research participants. However, there is potential for many harms to accrue at a group level in AI ^17^ since the technology capability is built on data from many individuals. It is therefore important to have group (community) involvement in AI research, to ensure that collective wellbeing, values and interests are recognized and respected ^18^ and societal concerns, inequities and other harms are addressed or avoided.

Additionally, the emerging use of public and private data for some AI applications, like machine learning, in part precipitated a shift from earlier AI systems development practices that were mainly technically-focused to now also being socially focused. Birhane *et al*. (2022) note that the initial cycles of AI research were not participatory in nature but logic-based; however, the focus on data-driven applications like machine learning prompted the involvement of non-experts in the construction of AI systems ^19^. Applying machine learning tools also prompts use of participatory methods to mitigate the sociotechnical risks characterized by fundamental tensions, uncertainties, and conflicts inherent in sociotechnical systems ^20^. A sociotechnical system is a way of theorizing technology as existing at the intersection of human, social, organizational, and technical factors in an effort to understand and potentially design technology ^21^. Taken together, these considerations provide a compelling basis for inclusion of CE in AI research and development efforts in general, and in Africa in particular, where diverse communitarian traditions and values are deeply seated. Such efforts are needed to identify both the opportunities and the challenges for AI development in Africa.

### 3. Challenges facing community engagement for AI research in Africa

CE necessitates a relatively clear identification of the research community, to understand how they may be impacted by the research and to ensure adequate representation in participatory processes. Defining the research community in AI research is challenging because AI researchers can often make use of existing data (i.e., secondary use) and potentially forgo interaction with study participants ^22^. This leaves AI researchers with the ability to develop systems that can impact lives and cultures ^23, 24^ without meaningfully involving those impacted. In some AI technology development, such as for machine learning (ML), the community is also not narrowly defined. This may not be unique to ML but it is remarkably pronounced here given that the data used in ML may come from diverse sources including; clinical care or research, social media, commercial or government databases, and information published digitally across the internet. Complexity arises here in operationalizing CE in that for some sources of data, there may exist specific CE frameworks, guidelines or principles that AI researchers presumably ought to be aware of. For example, ML may use data from genomics research and it’s likely that CE guidelines for genomics research developed by various organizations, including for example by the Human Heredity and Health in Africa (H3Africa) consortium ^25, 26^, are not readily known to AI researchers. Other data sources used within AI development projects could also have their specific CE frameworks. This gives rise to the question of whether AI researchers should be expected to uncover and apply all possibly applicable CE frameworks across a diverse dataset, or whether alternative CE framework(s) specific to AI research might be needed. As Floridi (2021) elaborates, since a range of initiatives (data sources) contribute guiding principles or values to AI development or implementation, there is potential for either overlap or confusion of such principles.

To add further complexity, CE aims to ensure mutual understanding between the researcher and the community; however, a number of African communities, particularly those that are rural, still have lower levels of exposure to and education about technology and are challenged with comprehending the nature of, and concepts linked to AI research ^27^. This can make it difficult for the research teams to communicate with the community throughout the course of AI research and afterwards, when sharing relevant insights and information derived that may be considered ethically obligatory ^28^.

As noted, CE can help ensure mutual respect (between the researcher and the community) and this involves understanding the attitudes community members have towards the research and how the research affects community values, norms, and interests. Certain cultural and social perspectives in some African communities have been reported to challenge the adaptation of new technologies ^27^. If not carefully pursued, AI research can generate capabilities, such as automation of traditionally inter-personal health practices that threaten community values, aspirations, and realities in Africa ^29^. A common example is the use of AI chatbots as a substitute or supplement to healthcare workers having conversations with patients. Throughout the continent, research groups have been testing the efficacy of AI-powered chatbots to deliver health education and encourage self-management. A scoping review of 12 chatbots revealed that chatbots did increase prosocial health behaviors such as vaccine acceptance, HIV testing, access to health information but were limited in their linguistic diversity ^30^. This noted, in many African communities, healthcare is perceived not only as an efficient and effective way to become physically well, but also represents an important cultural value of community members aiding one another in exercising solidarity aimed at attaining wellbeing for everyone ^14^. If the principle of solidarity is not explicitly incorporated into the development and use of health AI chatbots, such efforts may be perceived as a threat to cultural practices and values and this may compromise not only the uptake of this capability, but also potentially the community’s acceptance of other AI-focused interventions in the future. 

Another typical aspect of CE is ensuring that the study community stands to benefit reciprocally from the research. This is in service of the principle of justice, whereby the benefits and burdens of research are reasonably and equitably distributed. However, experiences of poverty amongst many communities in Africa pose a hurdle to the accessibility of the products of AI research since they may be costly or require access to underlying technology capabilities that may not be readily available. As such, in reality, it can be challenging to achieve the goals of benefit sharing and to honor commitments to reciprocity through AI research and development. As Adams (2022) indicated, the benefits of AI have been relatively slow to materialize across much of the African continent ^31^. 

Inclusiveness is a key consideration in attaining fairness, and non-discrimination in research participation. However, data collection methods for AI that are characterized as ‘top-bottom’, missing communities that fall outside the formal lens ^32^. Data for AI therefore, tends to exclude informal workers many of whom are women ^33^. Bonnet *et al*. (2019) report that 92% of total female employment in Sub-Saharan Africa is informal ^34^. Moreover, women and girls in some African societies have been marginalized because of patriarchy ^35, 36^ and excluded in some contexts ^37, 38^. This exclusion could be exacerbated by AI's “top-bottom” approach. It is therefore important that CE in AI aims at the realization of the possibilities for exclusion and work towards reducing rather than worsening such marginalization in Africa. 

CE is also premised on the notion that communities should be involved in all the stages of research, right from conception. Conceptually, this can be grounded in the notion that community members are rational beings, capable of deciding which initiatives they should be involved in, and which best serve individual and collective wellbeing. However, much of the ‘global health’ research in Africa has been driven by funders and research institutions in the Global North who amplify the resources and opportunities available to researchers in the Global South, with significant external influence over the research agenda ^39^. Additionally, given that researchers from the Global North potentially have more AI expertise ^40^, their influence in the research will likely be more than that of researchers in the Global South. This leads to a possibility of not including the input of the local communities in setting the research agenda. This can potentially result in meeting the interests of powerful institutions while making subordinate those of the local community. 

### 4. Context-specific priorities for CE in AI health research in Africa

Communities should be involved in AI health research early to allow their expectations, fears, values, interests and concerns to be incorporated into the design, testing, and implementation process ^27^. This entails involving communities in decision making in AI systems that stand to impact them. This can be achieved through open and honest dialogue ^41^ with the communities in an approach that is more consultative than educative so that the community feels more as partners than learners. Involvement of social scientists on AI research teams whose focus is on the impact that the AI may have on basic human rights and collective social values, can be an important way to begin to facilitate engagement ^42^.

When a particular AI capability being developed is meant to serve a diverse community, engagement efforts should seek to incorporate diverse perspectives. For example, in addition to engaging those who are of older age, involving young people in AI research can be important. This will give mileage to AI development given that the majority of the African population is relatively young; a median age of approximately 19 years ^43^. Adams (2022) notes a rising dynamic youth population in Africa with the vigor to embrace emerging digital enterprise ^44^. This is supported by the recognition by Eke *et al*. (2023) of a wide adoption of mobile technology in Africa that gives an optimism for the acceptance of AI technology as well ^27^. The inclusion of African youth therefore positions the continent to ensure that AI developed today is relevant to the next generation of users. For example, in AI health research, including youth may necessitate creative efforts to obtain their insights about the development of health AI research. While there is an acknowledged gap on engaging youth in AI development, there is need for research on providing an enabling environment for ongoing digital cooperation to identify and address unmet needs in AI health research in this population. 

Other endeavours can include AI researchers partnering with the community to develop guidance documents and frameworks for AI, rooted in the local context. Policies and initiatives should be geared at addressing some technical challenges, for example, with internet access and how this limitation can affect the health research process. A key practical challenge to the uptake of AI is inadequate internet connectivity ^45^. As of 2021, only 43% of the African population had internet access ^46, 47^. Additionally, Africa itself exhibits an internal digital divide where most internet infrastructure is concentrated in South Africa, Morocco, and Egypt ^47, 48^. This suggests a potential for marginalization in AI development in the form of exclusivity and inequality of access ^48^. Adams (2022) suggests the development of local AI capacities, skills and infrastructure in digital data and computing. Although these kinds of activities are not part of the usual responsibilities of researchers in some contexts, in the African context, researchers should consider technological capacity building as part of their remit and build in, and anticipate time and other resource needs into research plans and budgets.

Communities should be engaged in AI research in ways that empower. This could in turn potentially mitigate differences in power, privilege, and positionality; and permit a voice to the marginalized ^49^. For example, the aforementioned interface of AI health research and gender equity must be given key consideration in Africa since the continent is reported to already have a digital divide that tracks gender lines ^41^. As a start, women and girls could be included as collaborators in health AI research projects, as experts with lived experience of health needs specific to their sex and gender. In addition, women and girls can be involved as experts with lived experience of gender-based marginalization. The inclusion of women and girls can also be part of an asset-based approach to CE where they can share the unique strengths and resilience of African women and girls.

Engagement processes could also help with identifying and integrating diverse local languages in AI research and development. According to Gwagwa (2020), in Africa (and other contexts) these languages are marginalized because technology is developed largely with globally dominant languages in focus ^33^. Marivate *et al*. (2020) note that despite the over 2,000 languages spoken in Africa, text in these languages is rarely used in the training of NLP applications and indeed, many of these languages have limited textual representations ^50^. Inclusion of local languages, with consultation and permission from local language communities could help advance fairness in AI development. This can ensure that the capabilities and accompanying benefits of NLP accrue more to local communities than to those who communicate in languages found predominantly in socio-economically and politically powerful communities. Engagement processes are vital as, in addition to equitably distributing the benefits of AI, also realizing concerns as some communities may not want their linguistic and cultural traditions captured and represented in artificial environments.

## Conclusions

AI is hoped to help decrease health gaps and improve health outcomes in Africa and globally. However, the process of developing AI in and for Africa faces challenges and ethical concerns. CE is a process through which target communities can participate in identification and mitigation of sociotechnical harms of AI research and development, and in ensuring equitable distribution of AI benefits. While a number of challenges to effective CE exist with some particular presentations in the African context, with appropriate mitigation measures taken, CE could enable more ethical conduct of AI research.

## Ethics and consent

Ethical approval and consent were not required.

## Data Availability

No data are associated with this article.
