# Supplementary material for: Community engagement for artificial intelligence health research in Africa
Source: Wellcome Open Res. 2025 Mar 20;10:158. [Version 1] doi: 10.12688/wellcomeopenres.23684.1 (PMC12402761; doi:10.12688/wellcomeopenres.23684.1)
Supplement: Peer review history

# Reviewer report: Reviewer response for version 1

**DOI:** 10.21956/wellcomeopenres.26128.r129347

**Published:** 2025-09-01

**Authors:** James Andrew Shaw

**Affiliations:** 1 University of Toronto, Toronto, Ontario, Canada

Thank you for the opportunity to review this very well-written article on the opportunities and challenges of community engagement (CE) for health-related AI technologies in Africa. The article does well to frame the importance of CE in the design of AI technologies, describing the unique features of African countries that demand a tailored approach. The article also provide core definitions as foundations for the paper; a minor note is that a citation could be provided for the definition of "community", although the authors do acknowledge this is a diversely understood concept. The challenges outlined represent the knowledge and experience of the authors, and present an illustrative range of key considerations for those seeking to reflect on and engage in CE for AI in African countries. This is a strong article that warrants indexing.

Is the review written in accessible language?

Yes

Are all factual statements correct and adequately supported by citations?

Yes

Are the conclusions drawn appropriate in the context of the current research literature?

Yes

Is the topic of the review discussed comprehensively in the context of the current literature?

Yes

Reviewer Expertise:

Health ethics, health policy, digital health, artificial intelligence ethics

I confirm that I have read this submission and believe that I have an appropriate level of expertise to confirm that it is of an acceptable scientific standard.

# Reviewer report: Reviewer response for version 1

**DOI:** 10.21956/wellcomeopenres.26128.r121125

**Published:** 2025-04-02

**Authors:** Mary Kasule

**Affiliations:** 1 Not Applicable, Independent Consultant in Bioethics Place: Kampala, Kampala, Central, Uganda

**Title: Community Engagement for Artificial Intelligence Health Research in Africa**

 Overall, this is a well-written paper about a topical emerging issue of Community Engagement (CE) for Artificial Intelligence (AI) health research in Africa with significant implications for bioethics and AI health research. The paper defines community engagement, discusses the justification for CE  for AI health research in Africa,  how communities could be engaged, identifies the challenges facing CE for AI health research in Africa, and draws context-specific recommendations for effective CE. The study can be considered to be of great social value as it contributes to the limited data on effective CE for AI health research in Africa which is necessary for trust and acceptance of AI health research in Africa.

**However, I have some minor suggestions below:**

 1.   Since the paper discussed how communities in Africa could be engaged in AI health research and how it ought to be conducted, it is important to mention the expert stakeholders that could participate in the CE processes cited in the literature reviewed since non-availability of expert stakeholders might be one of the challenges to development of ethical, inclusive, and impactful AI health research in Africa. I suggest the authors list the target experts and stakeholders who were cited in the literature reviewed and their role in tye CE process in the introduction section so it’s clear from the start. For example, community members like community leaders can foster trust, inclusivity, and meaningful participation. Other participants could include patients, caregivers, researchers and academics, bioethicists, government and policymakers, funders, civil society, pharmaceutical companies, etc.

 2.   Since the document review method, was used to generate findings,  I suggest the authors briefly describe the steps that were followed in the process. 

 Otherwise, the paper can be approved with the above minor revisions

Is the review written in accessible language?

Yes

Are all factual statements correct and adequately supported by citations?

Yes

Are the conclusions drawn appropriate in the context of the current research literature?

Yes

Is the topic of the review discussed comprehensively in the context of the current literature?

Yes

Reviewer Expertise:

My areas of research are  Bioethics and Research Ethics Administration

I confirm that I have read this submission and believe that I have an appropriate level of expertise to confirm that it is of an acceptable scientific standard.
